# Silk Sericin Enrichment through Electrodeposition and Carbonous Materials for the Removal of Methylene Blue from Aqueous Solution

**DOI:** 10.3390/ijms23031668

**Published:** 2022-01-31

**Authors:** Yansong Ji, Xiaoning Zhang, Zhenyu Chen, Yuting Xiao, Shiwei Li, Jie Gu, Hongmei Hu, Guotao Cheng

**Affiliations:** 1 State Key Laboratory of Silkworm Genome Biology, College of Sericulture, Textile and Biomass Sciences, Southwest University, Chongqing 400715, China; jys2020@email.swu.edu.cn (Y.J.); c618@email.swu.edu.cn (Z.C.); xyt3209@email.swu.edu.cn (Y.X.); lsw20020127@email.swu.edu.cn (S.L.); cheng_2001@swu.edu.cn (G.C.); 2 Key Laboratory of Sustainable Utilization of Technology Research for Fisheries Resources of Zhejiang Province, Zhejiang Marine Fisheries Research Institute, Zhoushan 316021, China; 2008uky@gmail.com (J.G.); huhm@zju.edu.cn (H.H.)

**Keywords:** silk sericin, hydrogel, carbonization, adsorption, methylene blue

## Abstract

The recycling and reuse of biomass waste for the preparation of carbon-based adsorbents is a sustainable development strategy that has a positive environmental impact. It is well known that a large amount of silk sericin (SS) is dissolved in the wastewater from the silk industry. Utilizing the SS instead of discharging it into the environment without further treatment would reduce environmental and ecological problems. However, effective enrichment of the SS from the aqueous solution is a challenge. Here, with the help of carboxymethyl chitosan (CMCS), which can form a gel structure under low voltage, an SS/CMCS hydrogel with SS as the major component was prepared via electrodeposition at a 3 V direct-current (DC) voltage for five minutes. Following a carbonization process, an SS-based adsorbent with good performance for the removal of methylene blue (MB) from an aqueous solution was prepared. Our results reveal that the SS/CMCS hydrogel maintains a porous architecture before and after carbonization. Such structure provides abundant adsorption sites facilitating the adsorption of MB molecules, with a maximum adsorptive capacity of 231.79 mg/g. In addition, it suggests that the adsorption is an exothermic process, has a good fit with the *Langmuir* model, and follows the intra-particle diffusion model. The presented work provides an economical and feasible path for the treatment of wastewater from dyeing and printing.

## 1. Introduction

In recent years, with the rapid development of the manufacturing and textile industry, water pollution caused by the effluent from dyeing processes has attracted widespread attention. The commonly used organic dyes, such as methylene blue (MB), are toxic and carcinogenic aromatic compounds. It is well known that the excessive intake of textile dye from wastewater can cause nausea, vomiting, difficulties in breathing, and other adverse effects in humans and animals [1].

Several techniques, including biodegradation [2], filtration [3], and photocatalyst degradation [4], have been applied for the removal of dyes from the wastewater. Among those techniques, adsorption removal of dyes by carbonaceous adsorbent, derived from natural biomass and industrial by-products, has the advantages of simple operation and low-cost [5].

Silk sericin (SS) is a globular protein wrapped on the surface of silk fibers, accounting for approximately 20–30% of the total cocoon weight [6]. In the commercial silk industry, the cocoon needs to be degummed to generate the soft silk filaments. It has been reported that 400,000 tons of dried cocoons are produced worldwide annually, with at least 50,000 tons (12.5%) of degumming wastewater containing unutilized sericin released into the environment [7]. Although it is known that discarding SS carelessly can result in serious environmental and ecological problems, the enrichment of SS from wastewater is difficult due to its low molecular weight and high solubility in water [8]. Therefore, the effective approach for silk sericin enrichment would have huge economic and social benefits.

Although electrodeposition is commonly used for alloy and composite coatings fabrication [9,10,11], such technique has received considerable attention in biopolymeric hydrogel preparation [12,13]. In comparison with other gelation techniques, including chemical and physical crosslinking strategies, electrodeposition offers several benefits such as (i) does not require the presence of a chemical crosslinker and is, therefore, favorable for biocompatible hydrogel preparation, (ii) controllable design and flexibility for hydrogel preparation [14,15,16,17,18].

Carboxymethyl chitosan (CMCS) is a derivative of chitosan but with enhanced water solubility [19]. Our previous work showed that CMCS hydrogel can be prepared from a CMCS solution via electrodeposition under low voltage [14]. Because both CMCS and SS molecules contain abundant polar groups, such as −NH_2_ and −COOH groups, the formation of hydrogen bonds between CMCS and SS molecules is possible. We, therefore, presume that SS can crosslink with CMCS via hydrogen bonds and then electrodeposit along with CMCS on the anode to generate the composite hydrogel (Figure S4).

In this work, the CMCS molecules were introduced into the SS solution for the preparation of SS/CMCS hydrogel by electrodeposition under a low voltage. The gelation mechanism was explored. Subsequently, the SS/CMCS hydrogel was freeze-dried and carbonized to prepare porous sericin-based carbon material (SC). The adsorption capacity of SC for the removal of the cationic dye, MB, under various experimental conditions such as MB initial concentration, initial pH, adsorption time, and adsorption temperature were investigated. Isotherms, kinetics, and thermodynamic studies were conducted to evaluate the mechanisms of MB adsorption onto SC. Although both Kwak and Hong et al. [20,21] reported a method for the preparation of SS-based activated carbon for the adsorption of dyes, both studies did not explore the technique for the SS enrichment from the solution. In addition, both Kwak and Hong’s work required an extraordinary amount of KOH for the creation of SS-based carbon with a porous structure, which raises the cost and threatens the environment.

## 2. Results and Discussion

### 2.1. Characterization of SS/CMCS Hydrogel and SC

#### 2.1.1. Determination of SS Content and Intermolecular Interactions in SS/CMCS Hydrogel

As shown in Table S1, the percentage content of SS in SS/CMCS hydrogel was 77.12 ± 3.48% as determined via the Bradford assay, indicating the SS was the major component of the prepared hydrogel.

The viscosity of the 8% SS solution, the 1% CMCS solution, and the 4% SS/0.5% CMCS mixture were tested to investigate the intermolecular interaction between SS and CMCS molecules. The results are shown in Table 1.

It has been reported that the viscosity of the mixture can be determined by the Arrhenius equation [22]:

(1) $$ln\;\eta_{m}=\phi_{1}ln\;\eta_{1}+\phi_{2}ln\;\eta_{2}$$

where *η_m_* is the viscosity of the mixture in theory (mpa·s), *η_i_* is the viscosity of the pure component *i* (mpa·s), and *ϕ_i_* is the volume fraction of component *i* (*v*/*v*).

According to Equation (1), the theoretical viscosity of the SS/CMCS mixture was 3.03 mpa·s, which was less than the experimental value obtained (3.72 mpa·s). The results implied that there is some interaction between the SS and CMCS molecules in the mixture [23]. Under a pH of 7.48 (the pH of the prepared SS/CMCS solution), the carboxyl group (pKa = 4.53 [24]) and the amino group (pKa = 5.93 [25]) of CMCS were deprotonated. Therefore, the CMCS molecules carry negative charges. In addition, the isoelectric point of SS molecules has been reported at around a pH of 4 [26], meaning the SS molecules carried negative charges and the electrostatic interaction might not be the major interaction between SS and CMCS molecules.

However, the functional groups with polarity such as the amino group and carboxyl group of both SS and CMCS may form hydrogen bonds that can lead to crosslinking among SS and CMCS molecules. Moreover, it has been reported that the formation of hydrogen bonds between the components of the mixture can increase the viscosity [27]. As such, we hypothesize that intermolecular hydrogen bonds are formed among the SS and CMCS molecules, making the two components crosslink with each other.

**Figure 1:**
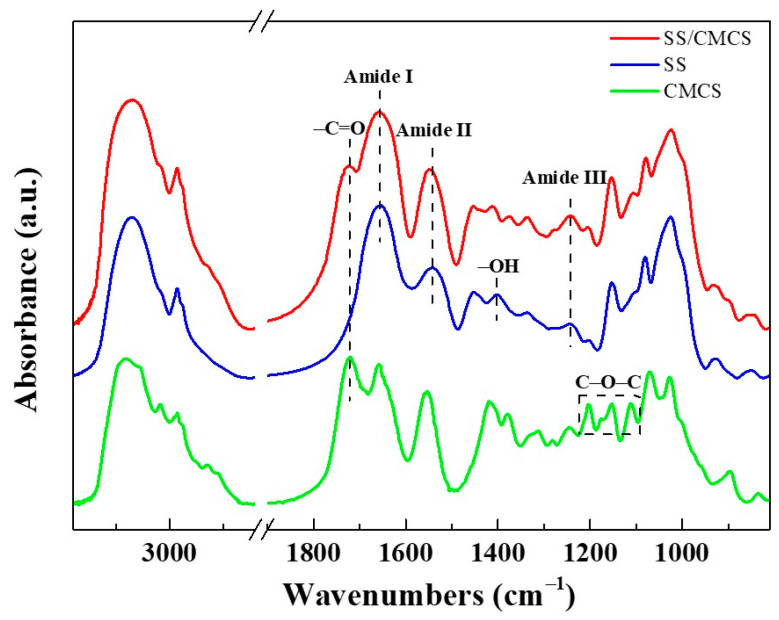
The FTIR spectra for freeze-dried SS solution, CMCS hydrogel and SS/CMCS hydrogel.

The FTIR spectra of the freeze-dried SS solution, the CMCS hydrogel, and the SS/CMCS hydrogel were recorded to explore the composition of the SS/CMCS hydrogel and the possible interaction between SS and CMCS molecules. As shown in Figure 1, the SS showed three characteristic adsorption peaks at 1655, 1541 and 1243 cm^−1^, which represented amide I, amide II and amide III, respectively [28].

The CMCS showed characteristic absorption bands at 1723 cm^−1^ (C=O double bond) [29], 1556 cm^−1^ (angular deformation of N−H bonds) [30], 1027 cm^−1^ (O−H bond stretching), and 1200 cm^−1–^1080 cm^−1^ (dissymmetry stretching vibrations of C–O–C in glycoside bonds) [31]. The FTIR spectrum of the SS/CMCS hydrogel contained the characteristic adsorption peaks of both SS and CMCS, indicating that the hydrogel was constituted by SS and CMCS.

In addition, compared to the SS, both amide I and amide II of SS/CMCS hydrogel shifted to higher wavenumber, from 1655 to 1659 cm^−1^ and 1541 to 1548 cm^−1^, respectively. As Płanecka et al. suggested, the shift of amide groups implied that there was intermolecular hydrogen bond formation [32]. Therefore, the observed shift was due to the formation of hydrogen bonds between SS and CMCS molecules.

#### 2.1.2. Evaluation of Thermal Stability

**Figure 2:**
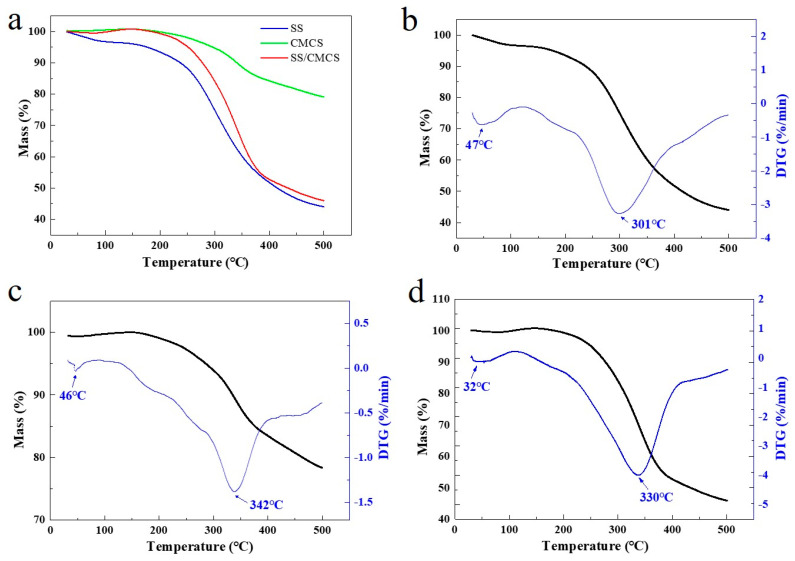
(**a**) TGA curves for the decomposition of freeze-dried SS solution, CMCS hydrogel, and SS/CMCS hydrogel; TGA-DTG profiles for freeze-dried (**b**) SS solution, (**c**) CMCS hydrogel, and (**d**) SS/CMCS hydrogel.

The thermal degradation behavior of SS, CMCS and SS/CMCS was evaluated by thermogravimetric analysis (TGA). From Figure 2a, it can be found that the TGA curves for both the freeze-dried SS/CMCS hydrogel and the SS solution have a similar type of spectral pattern. The likeness of the spectra can be attributed to the fact that the major component of SS/CMCS hydrogel is SS. It can be observed from Figure 2b–d that the derivative thermogravimetry (DTG) curves of all samples have an initial mass loss in the temperature range of 30–120 °C due to vaporization of the adsorbed moisture. The samples then experienced major, rapid mass losses between 120 and 500 °C, representing the thermal degradation via pyrolysis of the sample. It is known that the maximum thermal decomposition temperature (DTG_max_) corresponds to a temperature where thermal degradation occurs at the highest rate. DTG_max_ is considered an essential parameter to determine and compare the thermal stability of the sample [33]. The results showed that the DTG_max_ values of SS and CMCS were 301 and 342 °C, respectively. The DTG_max_ of the SS/CMCS hydrogel was 330 °C, indicating the thermal stability of SS/CMCS was lower than that of CMCS while higher than that of SS. The intermediate DTG_max_ temperature can be attributed to the introduction of SS, which reduced the crystallinity of CMCS.

#### 2.1.3. SEM Surface Morphology Characterization

**Figure 3:**
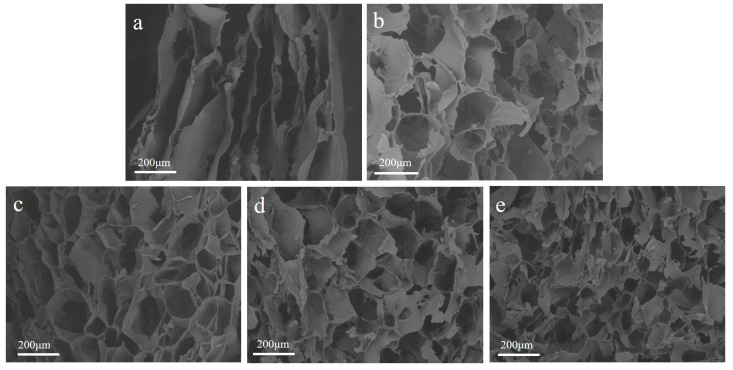
The SEM graphics of freeze-dried (**a**) CMCS hydrogel, (**b**) SS/CMCS hydrogel, and samples (**c**) SC850, (**d**) SC950, and (**e**) SC1050.

The SEM cross-sectional view of the freeze-dried CMCS hydrogel, the SS/CMCS hydrogel, and SC850, SC950, SC1050 are shown in Figure 3. It was found that the CMCS hydrogel displayed a multilayer structure, which was different from the porous structure of SS/CMCS hydrogel (Figure 3b). We believe the porous structure of the SS/CMCS hydrogel came from the intermolecular crosslinking among the SS and CMCS molecules. It can be seen from Figure 3c–e that the porous structure was retained after carbonization. As shown in Table 2, the pore size of the SCs tended to decrease as the carbonization temperature increased from 850 to 1050 °C; therefore, the SEM image of SC1050 exhibited a greater number of pores within the same size field of view, compared with the SEM images of SC850 and SC950.

#### 2.1.4. The Effect of Carbonization Temperature on Pore Development and Graphitization

**Figure 4:**
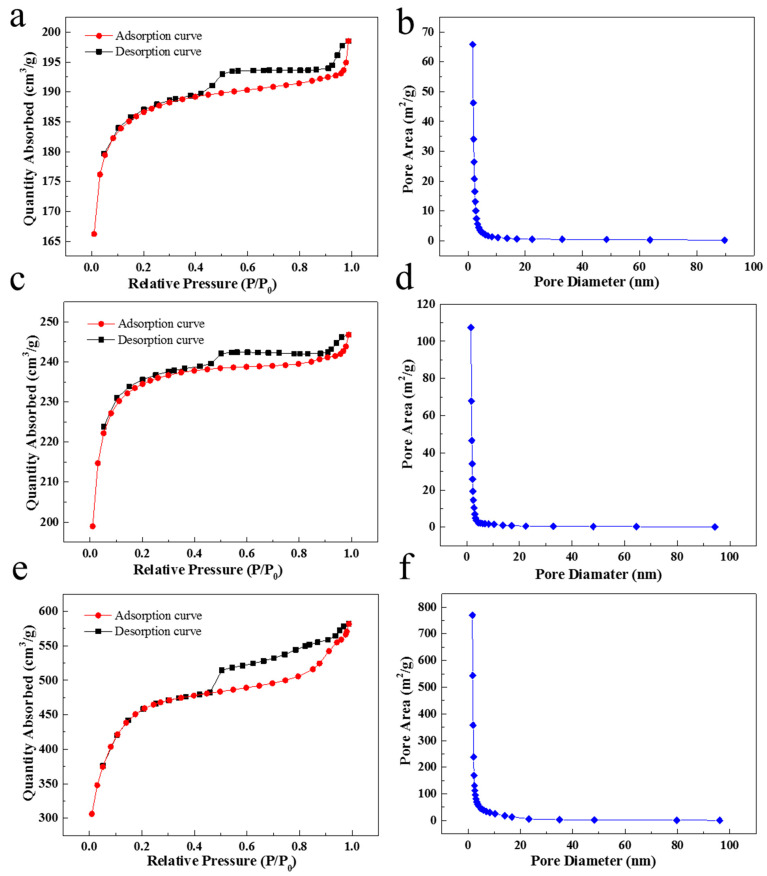
N_2_ adsorption–desorption isotherms and the pore size distributions of samples (**a**,**b**) SC850, (**c**,**d**) SC950 and (**e**,**f**) SC1050.

Nitrogen adsorption–desorption isotherms of SC850, SC950, and SC1050 are demonstrated in Figure 4a,c,e, respectively. As per IUPAC categorization, the shape of the isotherms from all SC samples were close to the type IV adsorption–desorption isotherm, which represented the physical adsorption of the typical mesoporous adsorbents [22]. In addition, the hysteresis loop of all SC samples belongs to type H4, which was often found in the activated carbon with narrow slit-like pores [34]. Furthermore, the Barrette–Joynere–Halenda (BJH) pore size distribution (Figure 4b,d,f) confirmed that the SC sample was mesoporous (2–50 nm) materials [22].

As shown in Table 3, with increased carbonization temperature, the specific surface area and the total pore volumes increased, while the BJH average diameter decreased. This is attributed to the higher carbonization temperature resulting in a greater amount of volatiles being released from the SC sample, creating higher micropore volume [35]. As the molecular size of MB is 1.43 × 0.61 × 0.4 nm [36], which is smaller than the BJH average diameters of SCs, the MB molecule can enter the mesoporous structure with ease.

**Figure 5:**
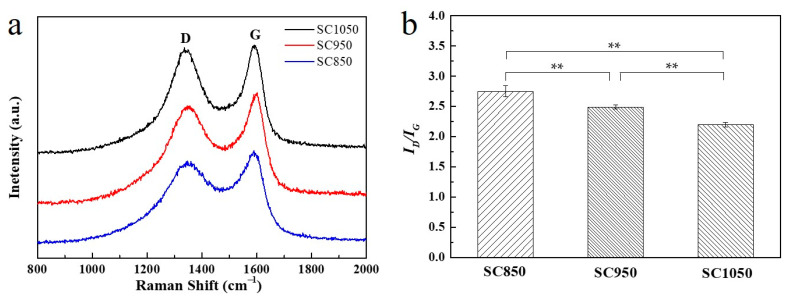
(**a**) Raman spectra and (**b**) calculated *I_D_/I_G_* values for samples SC850, SC950 and SC1050. (n = 3, ** *p* < 0.01).

As shown in Figure 5a, the Raman spectra of the SC samples displayed two characteristic peaks at 1340 (*D* band) and 1590 cm^−1^ (*G* band). The *D* band represents the defects and disorder in the carbon lattice, and the *G* band represents the in-plane stretching vibration of sp^2^ hybrid carbon atoms in graphite layers. Together, the *D* and *G* bands provide spectroscopic evidence for the presence of graphite in the sample [37]. The graphitization degree can be determined by the integrated intensity ratio of *D* and *G* bands (the deconvoluted Raman spectra are displayed in Figure S6), *I_D_/I_G_*, and a lower *I_D_/I_G_* value generally indicated a higher graphitization degree [37].

Figure 5b shows that SC1050 had a higher graphitization degree compared to that of SC850 and SC950. The higher degree of graphitic structure is beneficial for the adsorption of dye molecules with an aromatic ring because it promotes the π–π interaction between the adsorbent and the dye molecule [38].

#### 2.1.5. Cytotoxicity Evaluation

**Figure 6:**
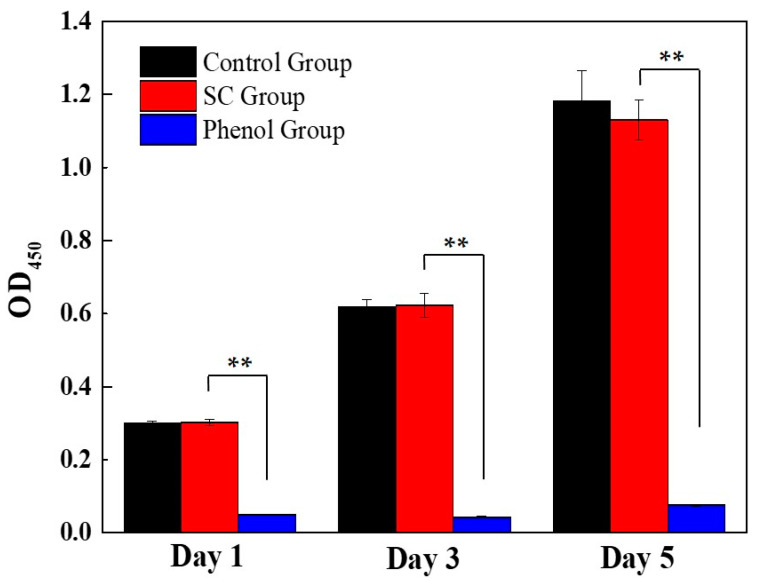
Toxicological effect of SC1050 on HEK-293 cells. (n = 3, ** *p* < 0.01).

The cytotoxicity of the SC1050 was evaluated to ensure that its usage would not cause health problems. The phenotype of HEK-293 cells for the control group, the SC1050 group, and the phenol group are shown in Figure S7. As depicted in Figure 6, the positive control group demonstrated a significantly lower OD_450_ value when compared with that of the control group and the SC1050 group. However, there was no statistically significant difference between the OD_450_ values of the control group and the sample group, indicating that SC1050 is not toxic.

### 2.2. Dye Adsorption Experiments

#### 2.2.1. Effect of MB Initial Concentration

**Figure 7:**
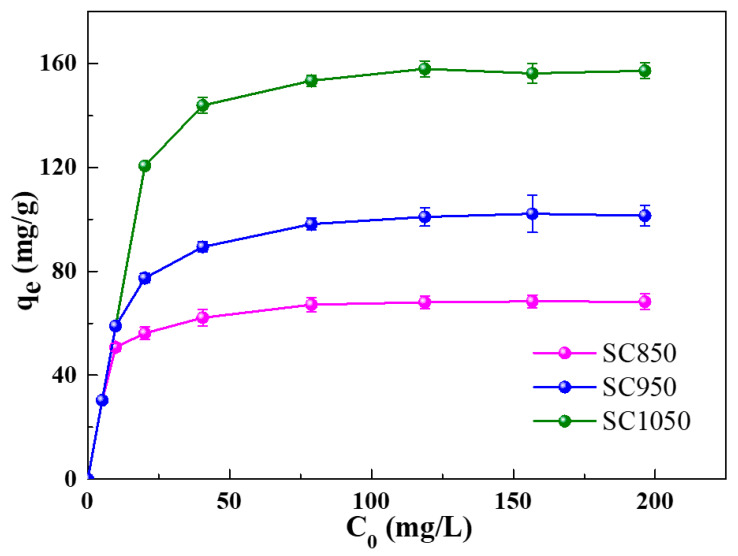
Effect of initial MB concentration on the adsorption process.

The initial dye concentration provides an important driving force to enhance the adsorption properties of the adsorbents for the dye molecules [39]. The experiment was therefore conducted at the initial MB concentrations of 5, 10, 20, 40, 80, 120, 160 and 200 mg/L. The pH of the mixture was adjusted to 7 and was maintained at 25 °C in an incubator operated at a speed of 150 rpm. As shown in Figure 7, when the initial concentrations of MB in solution were within the range of 5–80 mg/L, the adsorption capacity of all SC samples enhanced with a rise in initial MB concentration. The results can be attributed to the driving force of the increased initial MB concentration, which helped to overcome the mass transfer resistance of MB between the aqueous and the solid phase [40]. As shown in Figure S8, the color of the MB solution turned transparent or light blue after the adsorption experiment. From Figure 7, it was found that the saturation was not reached at MB initial concentration of 80 mg/L, such initial concentration was therefore selected for the subsequent experiments.

When the MB initial concentration reached and exceeded 120 mg/L, the *q_e_* of SC samples remain unchanged, indicating that the surface sites of the SC sample for adsorption were fully occupied by MB molecules. In addition, it was found from Figure 7 that the adsorption capacity of the SC samples increased as the carbonization temperature increased. Such result can be attributed to SC1050 having the largest specific surface area and the highest graphitization degree of SC1050, as determined by the nitrogen adsorption–desorption isotherms and Raman spectroscopy, respectively.

Furthermore, it has been widely reported that π–π stacking interactions play a crucial role in the adsorption of MB molecules on carbonous adsorbent [41,42]. In addition, the increase in the degree of graphitization would promote such adsorption behavior due to the escalated sites for π–π stacking interactions [43,44]. Therefore, it could be proposed that the mechanism for the adsorption of MB molecules on the SC samples involves π–π stacking interactions and, from our results, that the degree of graphitization and adsorption capacity of SC samples increased with the rise of carbonization temperature.

#### 2.2.2. Effect of Solution Initial pH

**Figure 8:**
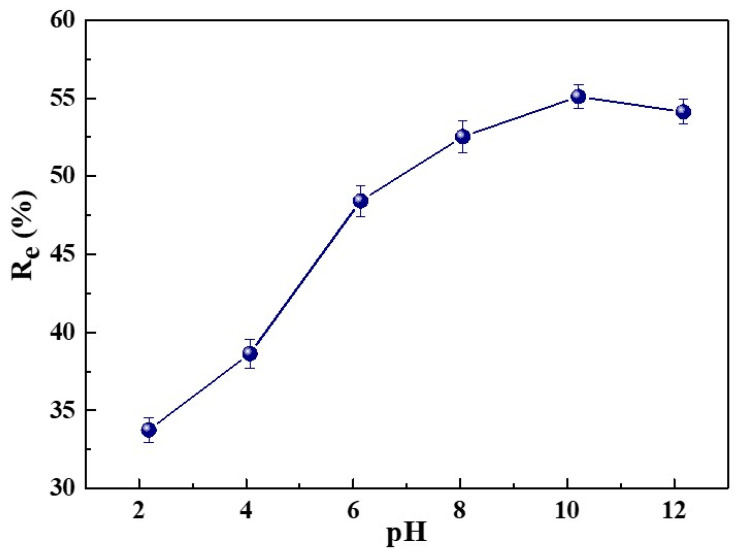
The effect of the solution’s initial pH on *R_e_* of MB by SC1050.

Solution pH plays an important role in the adsorption process because the solution pH can affect the surface charge of the adsorbent and the degree of ionization of dye molecules [39]. The impact of the solution’s initial pH was explored by adding 5 mg of SC1050 into the MB solution, with an initial concentration of 80 mg/L, at an initial pH of 2, 4, 6, 8, 10 or 12. The adsorption experiment was carried out at a temperature of 25 °C in an incubator with a shaking speed of 150 rpm. As shown in Figure 8, the adsorption capacity of SC1050 increased dramatically as the solution initial pH increased from 2 to 10, where *R_e_* increased from 33.76 ± 0.80% to 55.11 ± 0.75%. Because pH_pzc_ of SC1050 was 7.3 (Figure S9) at pH lower than pH_pzc_, the SC1050 surface was positively charged. The repulsion between the cationic dye molecule, MB in our case, and the positively charged surface would lead to a decrease in MB adsorption. At pH higher than pH_pzc_, the SC1050 surface was negatively charged, promoting the adsorption of cationic MB molecules. However, when the solution pH was greater than 10, a slight decrease in adsorption capacity of SC1050 can be observed, which can be attributed to the poor stability of MB at high pH (Figure S10 exhibits the peak position shift of the UV-Vis spectrum of MB at pH of 12) [45], rather than the adsorption capacity decrease in SC1050.

#### 2.2.3. The Effect of Adsorption Temperature and the Study for Adsorption Thermodynamics

**Figure 9:**
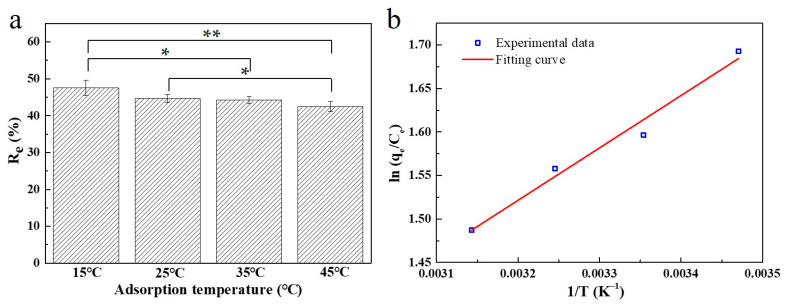
(**a**) The effect of adsorption temperature for the adsorption of MB onto SC1050 and (**b**) the plot for estimation of thermodynamic parameters. (n = 3, * *p* < 0.05, ** *p* < 0.01).

To explore the effect of temperature on adsorption behavior of SC1050, 5 mg of SC1050 was added into MB solution at an initial concentration of 80 mg/L with an adjusted pH of 10. The test was performed at temperatures of 15, 25, 35 and 45 °C in an incubator with a shaking speed of 150 rpm. As shown in Figure 9a, when the temperature increased from 15 to 45 °C, the value of *R_e_* decreased from 47.53 ± 2.04% to 42.44 ± 1.36%, indicating that the adsorption process was more favorable at low temperature.

The thermodynamic parameters, including ∆*G*, ∆*H*, and ∆*S*, of the adsorption process were determined using the Van’t Hoff equation. The fitting curve is displayed in Figure 9b and the calculated thermodynamic parameters are given in Table 4. The negative values of ∆*H* indicated that adsorption was an exothermic process. The negative value of ∆*S* indicated that the randomness at the solid–liquid interface decreased during the adsorption process [46].

The Gibbs free energy change (∆*G*) was calculated from Equation (2):

(2) ΔG=ΔH−TΔS

where, *T* is the adsorption temperature (*K*), ∆*H* is the enthalpy change (kJ·mol^−1^), and ∆*S* is the entropy change (J·mol^−1^·K^−1^).

The negative value of ∆*G* indicated the adsorption process was spontaneous. Moreover, it is known that Δ*G* values for the physisorption are in the range of −20 to 0 kJ/mol, for the physisorption together with chemisorption are in the range of −20 to −80 kJ/mol, and for the chemisorption are in the range of −80 to −400 kJ/mol [47]. As shown in Table 4, the values of ∆*G* for MB adsorption from 288.15 to 318.15 K were all in the range of −20–0 kJ/mol, which indicated that the adsorption process favored for a physisorption mechanism.

#### 2.2.4. The Effect of Adsorption Time and the Kinetics of Adsorption

**Figure 10:**
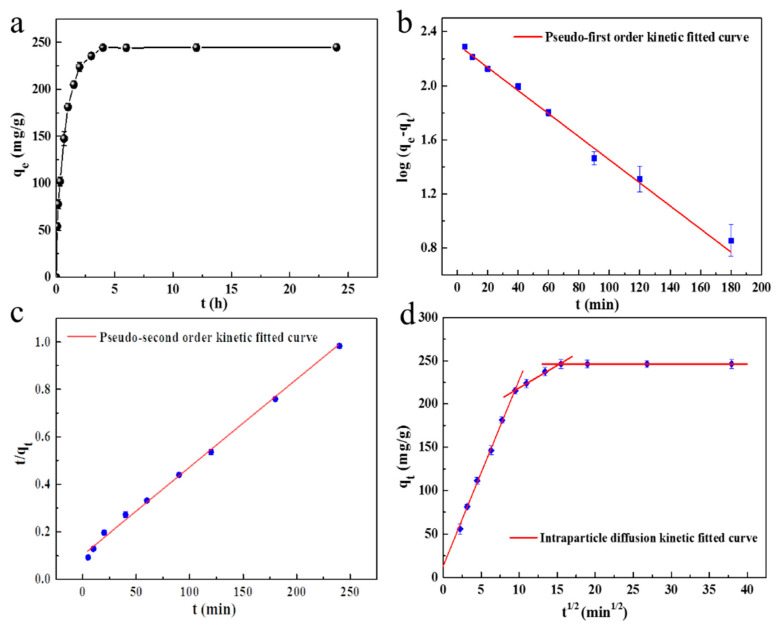
(**a**) The effect of adsorption time for the adsorption of MB onto SC1050 and plots for (**b**) pseudo-first-order, (**c**) pseudo-second-order and (**d**) intra-particle diffusion models.

To study the effect of adsorption time on the MB adsorption, 5 mg of SC1050 was added into the MB solution at an initial concentration of 80 mg/L (with an adjusted pH of 10). The test was performed at a temperature of 15 °C in an incubator with a shaking speed of 150 rpm. As shown in Figure 10a, the initial adsorption was observed to be very fast, due to the availability of abundant vacant adsorption sites. The adsorption decreased significantly within two hours to give a gradual approach to an equilibrium, which was attributed to the saturation of adsorption sites or a pore diffusion process of the MB molecule. Finally, the adsorption reached a plateau after 4 h incubation. Furthermore, the curves obtained were single, smooth, and continuous, which suggested the possibility of MB monolayer coverage onto the surface of SC1050 [48].

The kinetic data of adsorption was analyzed using pseudo-first-order (Figure 10b), pseudo-second-order (Figure 10c) and intra-particle diffusion (Figure 10d) models. As shown in Table 5, the correlation coefficient R^2^ of the intra-particle diffusion model was the highest, which suggested that the adsorption mechanism was predominantly intra-particle diffusion. In Figure 10d, the intra-particle diffusion curve exhibited multilinear plots. Three steps can be identified from the intra-particle diffusion curve, which indicated that there are three processes that affect the adsorption. The first process corresponded to the external diffusion adsorption of MB molecules on SC1050; the second represented the diffusion of MB molecules into the inner structure of SC1050; and the third was attributed to the equilibrium stage. Moreover, the C_i_ was not equal to zero, which implied that the intra-particle diffusion was not the only rate-limiting step [49].

#### 2.2.5. Study of Adsorption Isotherms

**Figure 11:**
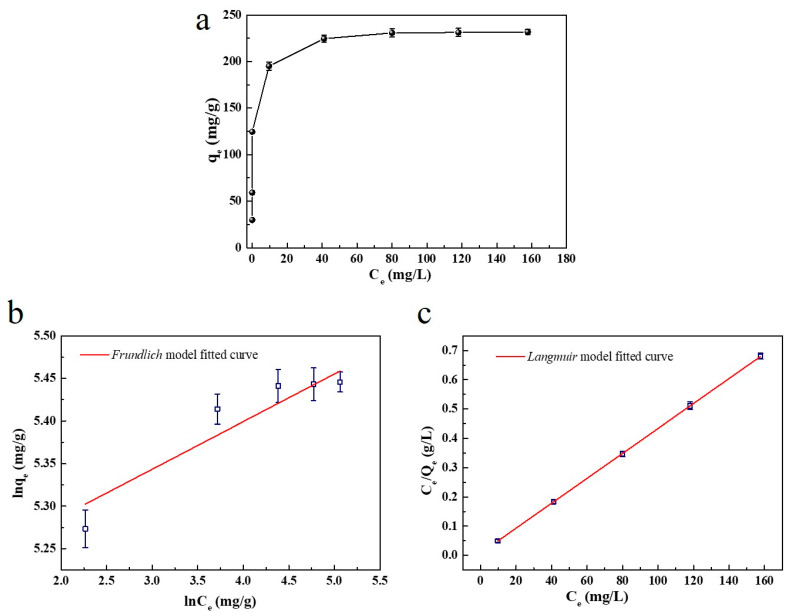
(**a**) Adsorption isotherm, (**b**) *Langmuir* fitting and (**c**) *Frundlich* fitting.

The adsorption isotherms describe the relationship between the quantity of adsorbate and the equilibrium concentration of the adsorptive, which is important information that can be used to understand the adsorption process [50]. Here, 5 mg of SC1050 was added into MB solution at the initial concentration of 5, 10, 20, 40, 80, 120, 160 or 200 mg/L (with the pH adjusted to 10). The test was performed at 15 °C in an incubator with a shaking speed of 150 rpm. It can be found from Figure 11 that the *q_e_* increased to 231.79 mg/g with a raised MB initial concentration. This result was consistent with the study in Section 2.2.1, which shows that the higher initial concentration would enhance the adsorption process. The results were then analyzed by *Langmuir* and *Freundlich* isotherm models. The linear plot showed that the adsorption obeyed the *Langmuir* isotherm model with a greater value of R^2^ value than that of the *Freundlich* adsorption model (Table 6). Since the adsorption followed the *Langmuir* adsorption, it can be suggested that the adsorption of the MB molecule onto the SC1050 surface corresponded to a monolayer without lateral interaction among the adsorbed molecules, which was consistent with the results obtained from Section 2.2.4. The maximum monolayer adsorption amount calculated (q_max,cal_) from the *Langmuir* isotherm model was 234.72 mg/g. Furthermore, the R_L_ value of the *Langmuir* model was greater than 0 but less than 1, which suggested favorable adsorption behavior of MB onto SC1050 [1].

## 3. Materials and Methods

### 3.1. Chemicals

Carboxymethyl chitosan (CMCS) was purchased from Shanghai Ryon Biological Technology CO., Ltd. (Shanghai, China). Silk sericin (SS) was from Langzhong Silkworm Breeding Farm of Sichuan Province (Sichuan, China). Sodium chloride was purchased from Aladdin Biotech CO., Ltd. (Shanghai, China). Potassium bromide was purchased from Sango Biotech Co., Ltd. (Shanghai, China). Bradford assay kit was purchased from Beyotime Biotechnology Co., Ltd. (Shanghai, China). Sodium hydroxide was purchased from Jinshen Chemical Test Co., Ltd. (Chengdu, China). Hydrochloric acid was purchased from Chuandong Chemical Co., Ltd. (Chongqing, China). Cell Counting Kit-8 (CCK-8) was purchased from Mei5 Biotechnology Co., Ltd. (Beijing, China). Trypan blue was purchased from Macklin Biochemical Co., Ltd. (Shanghai, China). The filter membrane with a pore size of 0.22 μm was purchased from Jiangsu Green Union Science Instrument Co., Ltd. (Jiangsu, China). All chemicals were of analytical grade and were used without further purification. Deionized water was obtained from a Milli-Q Direct-8 purification system (resistivity >18 MΩ·cm, Millipore Inc., Boston, MA, USA) onsite and was used in all experiments.

### 3.2. Preparation of Sericin Derived Carbon (SC)

#### 3.2.1. Fabrication of SS/CMCS Hydrogel

First, the pH of the CMCS solution was adjusted to 12 with 5 M of sodium hydroxide solution. Then, an 8% (*w*/*v*) SS solution was mixed with the CMCS solution of a certain concentration for 4 h at 25 °C. It has been reported that low-temperature benefits the formation of hydrogen bonds among the components in solution [51]. Therefore, to facilitate the formation of hydrogen bonds between SS and CMCS molecules, the prepared SS/CMCS mixture was kept in a refrigerator at 4 °C overnight.

An electrochemical workstation (CHI760E, Shanghai Chenhua Instruments Co., Ltd., Shanghai, China) and a three-electrode assembly were used for the SS/CMCS hydrogel fabrication, in which a graphite electrode, an Ag/AgCl electrode, and a platinum electrode were utilized as the working, reference, and counter electrode, respectively.

To fabricate the SS/CMCS hydrogel, the electrodes were immersed in the SS/CMCS aqueous solution, containing 0.5% (*w*/*v*) NaCl, and a voltage in direct current (DC) mode was applied by an amperometric I-t curve method. The generated hydrogel was gently peeled from the graphite electrode and then carefully rinsed three times with deionized water for 15 min to remove any residue that remained. The gelation parameters were optimized and described in Section 1 of Supplementary Materials.

#### 3.2.2. Carbonization of the SS/CMCS Hydrogel

The prepared SS/CMCS hydrogel was freeze-dried by using a lyophilizer (LGJ-10, YuMing Instrument Co., Ltd., Shanghai, China). Then, the freeze-dried hydrogel was carbonized in a tube furnace (OTF-1200X-S, Hefei KeJing Materials Technology Co., Ltd., Hefei, China) using the following heating schedule: the samples were first heated from room temperature to 150 °C at a rate of 10 °C/min and kept for 120 min in a N_2_ flow to remove the water absorbed on the sample; the sample was then heated to 350 °C at a rate of 5 °C/min and kept for 180 min; then, the temperature was increased from 350 to 850, 950, or 1050 °C at a rate of 2 °C/min and maintained for 120 min to induce the formation of graphitic structures [52]. Subsequently, the sample was rinsed with deionized water heated to 60 °C until the pH of the rinse water was neutral. The samples were then frozen dried and stored in desiccators for later use. According to the carbonization temperature, the prepared samples were named as SC850, SC950, and SC1050, respectively.

### 3.3. Characterization

#### 3.3.1. Evaluation of SS Content in SS/CMCS Hydrogel

The mass percentage of SS (*n*%) in the SS/CMCS hydrogel was investigated based on the Bradford assay [10]. First, 1% CMCS solution was prepared and recorded as solution A. From solution A, the CMCS hydrogel was prepared according to the procedure described in Section 2.2.1. The remaining CMCS solution was recorded as solution B. Then, 40%, 20%, 10%, 5%, 2.5%, 1.25% and 0.625% (*w*/*v*) SS solution were mixed with solution A and B, with a volume ratio of 1:1, respectively. After being diluted eight times, 5 μL of the above specimen were mixed with 250 μL of Brilliant Blue G-250 dye for the determination of SS concentration and to obtain the standard curve of SS in a different matrix, before and after the gelation process (Figure S5). The measurements were performed using a microplate reader (Synergy H1, Bio-Tek, Winooski, VT, USA) at 595 nm.

The mass percentage of SS in SS/CMCS hydrogel was then calculated using Equation (3):

(3) $$n\%=\frac{C_{1}V_{1}-C_{2}V_{2}}{m}\times 100\%\;$$

where *C*_1_ and *C*_2_ are the concentrations (mg/mL) of SS in the SS/CMCS mixture before and after the gelation; *V*_1_ and *V*_2_ are the volumes (mL) of the SS/CMCS mixture before and after the gelation; *C*_1_*V*_1_ and *C*_2_*V*_2_ represent the mass (mg) of SS in the SS/CMCS mixture before and after gelation; (*C*_1_*V*_1_ − *C*_2_*V*_2_) represents the mass (mg) of SS in the SS/CMCS hydrogel; *m* is the weight (mg) of freeze-dried hydrogel.

#### 3.3.2. Viscosity Evaluation

The viscosity of the 8% SS solution, 1% CMCS solution, and the mixture containing 4% SS and 0.5% CMCS (4% SS/0.5% CMCS) were evaluated using a viscometer (DV3TLVCJ0, Brookfield, USA) with a 48 mm parallel plate and a shear rate of 50 rpm at 25 °C. All samples were kept in viscometer for 5 min to ensure temperature equilibration.

#### 3.3.3. Fourier-Transform Infrared (FTIR) Spectroscopy Analysis

The FTIR of the freeze-dried SS solution, as well as the SS/CMCS and CMCS hydrogels were analyzed through the KBr disc method, using an infrared spectrometer (Nicolet iN10, Thermo Fisher Scientific, USA). The absorption spectra of samples in the region (4000–400 cm^−1^) were acquired with 24 scans at a resolution of 4 cm^−1^.

#### 3.3.4. Thermogravimetric (TGA) Analysis

The thermal stability of the freeze-dried SS solution, as well as the SS/CMCS and CMCS hydrogels were studied by a thermal analyzer (STA 449 F3 Jupiter, Netzsch, Germany). The samples were heated from 30 to 500 °C, under N_2_ atmosphere, at a constant heating rate of 10 °C/min.

#### 3.3.5. Scanning Electron Microscopy (SEM) Surface Morphology Characterization

First, the samples were sputter-coated (JS-19158, KYKY Technology CO., LTD, China) with gold. The micromorphology of the freeze-dried SS/CMCS hydrogel, CMCS hydrogel, and SC850, SC950, SC1050 were observed using a SEM (TM4000Plus, Hitachi, Tokyo, Japan). The pore size distribution of each sample was analyzed by measuring the length (*L*) and width (*D*) of the pore structure using Image J (V1.8.0, National Institutes of Health, USA). The average pore diameters (*d*) were calculated by Equation (4).

(4) $$d=\frac{L+D}{2}$$

#### 3.3.6. BET-BJH Analyses

N_2_ adsorption–desorption isotherms of all SC samples were tested by a physical absorbent analyzer (ASAP 2460, Micromeritics, Norcross, GA, USA). The specific surface area of all SC samples were determined by the Brunauer–Emmett–Teller (BET) method. The pore volume and the diameter distribution were calculated by the BJH method.

#### 3.3.7. Raman Spectrum Measurement

The Raman spectra of the SC850, SC950, SC1050 in the region of 3500–100 cm^−1^ were acquired by a spectrometer (Renishaw inVia, Renishaw, New Mills, UK) at a resolution of 2 cm^−1^ and an excitation wavelength of 532 nm. All data were processed using the Origin software (Origin Pro 9.0, OriginLab Corp., Northampton, MA, USA). Following the method reported by Salinas et al. [53], the Lorentz function was used to fit *D* and *G* bands of Raman spectra. The integral area ratio (*I_D_/I_G_*) between the *D* band and *G* band was calculated from the fitted data of Raman spectra. 

#### 3.3.8. Determination of Zero Point Charge (pH_pzc_)

In order to reveal the mechanism of adsorption, the pH_pzc_ of the sample was determined using the following procedure [54]: 10 mL of 0.01 M NaCl were adjusted to the initial pH (pH_initial_) value of 2, 4, 6, 8, or 10 using 0.1 M HNO_3_ or 0.1 M NaOH and a pH meter (PHS-3E, Shanghai INESA Scientific Instrument Co., Ltd., Shanghai, China) in weighing bottle. A total of 5 mg of SC1050 was then added into each weighing bottle, which were placed in a shaker at 25 °C for 48 h with a shaking speed of 150 rpm. The final pH (pH_final_) of the specimen was measured and plotted against the pH_initial_ values. The pH_pzc_ of SC1050 was found at the intersection between the pH_final_–pH_initial_ curve and the diagonal straight line.

#### 3.3.9. Cytotoxicity Assay

The cytotoxicity of SC1050 was evaluated via cell counting kit-8 (CCK-8) using human embryonic kidney 293 (HEK293) cells [55]. Specifically, 0.2 g of SC1050 was immersed in 10 mL of Dulbecco’s Modified Eagle Medium (DMEM) for 72 h at 37 °C. The mixture was then filtered through a 0.2 µm sterile syringe filter to obtain the leaching liquor. Subsequently, HEK-293 cells were seeded in a 96-well plate at a concentration of 5 × 10^3^ cells/well in 100 µL of complete growth medium (90% DMEM with 10% FBS) and were cultured for 24 h at 37 °C in a humidified 5% CO_2_ atmosphere. Then, 10 µL of complete growth medium (negative control), phenol (64 g/L, positive control), and the leaching liquor were added to each well and incubated. The medium of each group was replaced with fresh medium at a 24 h interval. CCK-8 assays were conducted after 1, 3, and 5 days of incubation. The absorbance was determined at 450 nm using a microplate reader (Synergy H1, Bio-Tek, Winooski, VT, USA).

### 3.4. Dye Adsorption Behavior of SC Samples

The water-soluble cationic dye, MB, was selected as the model contaminant. The effect of MB initial concentration, initial pH, adsorption time, and adsorption temperature on the MB removal were investigated below. The concentration of MB in the solution was monitored by an ultraviolet-visible (UV-Vis) spectrophotometer (UV1900, Shanghai Jinghua Technology Instrument Co., Ltd., Shanghai, China) at 664 nm.

#### 3.4.1. The Effect of MB Initial Concentration

A total of 5 mg of SC sample was added into 30 mL of MB solution with concentrations of 5, 10, 20, 40, 80, 120, 160 and 200 mg/L. For the adsorption experiment, the mixture was placed in an incubator with a shaking speed of 150 rpm for 24 h at 25 °C. Then, the mixture was filtered with a 0.22 µm pore size membrane, and the absorbance of the solution was measured by UV-Vis.

The percentage removal (*R_e_*) and the amount of equilibrium adsorption, *q_e_* (mg/g), can be calculated using Equations (5) and (6), respectively.

(5) $$R_{e}=\frac{\left(C_{0}-C_{e}\right)}{C_{e}}\times 100\%$$

(6) $$q_{e}=\frac{\left(c_{0}-c_{e}\right)\times V}{M}$$

where *C*_0_ and *C_e_* are the initial and equilibrium concentrations of MB in solution (mg/L); M is SCs dosage (mg); V is the volume of MB solution (mL).

#### 3.4.2. The Effect of Initial pH

The pH of MB solution was adjusted to 2, 4, 6, 8, 10, and 12 by using 1 M of HCl or NaOH and a pH meter. Then, 5 mg of SC1050 was added to the 30 mL of MB solution with an initial concentration of 80 mg/L for the adsorption experiment described in Section 3.4.1. The concentration of MB that remained in the solution was measured by UV-Vis.

#### 3.4.3. The Effect of Adsorption Temperature and Adsorption Thermodynamics

A total of 5 mg of SC1050 was added into 30 mL of MB solution with an initial concentration of 80 mg/L. The mixture was placed in an incubator with a shaking speed of 150 rpm at 15, 25, 35, and 45 °C for 24 h. After filtration, using the procedure described in Section 3.4.1, the concentration of MB remaining in the solution was measured by UV-Vis. To better understand the effect of the adsorption temperature on the adsorption process, the thermodynamic parameters were determined using the Van’t Hoff equation [56].

(7) $$\ln\left(\frac{q_{e}}{C_{e}}\right)=\frac{-\Delta H}{RT}+\frac{\Delta S}{R}$$

where *R* is the gas constant (8.314 J·mol^−1^·K^−1^), *T* is the adsorption temperature (K), Δ*H* is the change in enthalpy (kJ·mol^−1^) and Δ*S* is the change in entropy (J·mol^−1^·K^−1^).

#### 3.4.4. The Effect of Adsorption Time and Adsorption Kinetics

For the adsorption experiment, 5 mg of SC was added into 30 mL of MB solution with a concentration of 80 mg/L. The concentration of MB that remained in the solution was determined at 5 min, 10 min, 20 min, 40 min, 1 h, 1.5 h, 2 h, 3 h, 4 h, 6 h, 12 h, and 24 h by UV-Vis. The amount of adsorption at time *t* can be calculated by Equation (8):

(8) $$q_{t}=\frac{\left(C_{0}-C_{t}\right)}{M}\times V$$

where *q_t_* is the amount of adsorption at time t (mg/g), and *C_t_* is the MB concentration at time t (mg/L).

The pseudo-first-order, pseudo-second-order, and intra-particle diffusion models were used for the adsorption kinetic study in order to understand the adsorption rate, the time required for reaching equilibrium, and the mass transfer mechanisms of the adsorption system, respectively.

The pseudo-first-order model was described by Equation (9) [57]:

(9) $$log\left(q_{e}-q_{t}\right)=logq_{e}-\frac{k_{1}t}{2.303}$$

where *t* is the adsorption time (min), and *k*_1_ is the pseudo-first-order rate constant (min^−1^).

The pseudo-second-order model was described by Equation (10) [57]:

(10) $$\frac{t}{q_{t}}=\frac{1}{k_{2}q_{e}^{2}}+\frac{1}{q_{e}}t$$

where *k*_2_ is the pseudo-second-order rate constant (g·mg·min^−1^).

The intra-particle diffusion model was described by Equation (11) [58]:

(11) $$q_{t}=k_{i}t^{1/2}+C_{i}$$

where, *k_i_* is the intra-particle diffusion rate constant (mg·g·min^−1/2^) and *C_i_* is the boundary layer effect. When the value of *C_i_* is zero, intra-particle diffusion is the sole rate-limiting step. If *C_i_* does not equal zero, the intra-particle diffusion is not the only rate-limiting step.

#### 3.4.5. Adsorption Isotherm

The adsorption capability of SC1050 on MB under the optimum conditions was studied by adding 5 mg of SC1050 to 30 mL of the MB solution (pH = 10) with concentrations of 5, 10, 20, 40, 80, 120, 160, and 200 mg/L. Isotherm data were analyzed using the equilibrium models of the *Langmuir* isotherm and the *Freundlich* isotherm. Among them, the *Langmuir* isotherm is based on the monolayer adsorption on homogeneous surfaces, while *Freundlich* isotherm is commonly used to describe multilayer adsorption on heterogeneous surfaces [59].

The *Langmuir* isotherm is described below [60]:

(12) $$\frac{C_{e}}{q_{e}}=\frac{1}{q_{max}\cdot K_{L}}+\frac{C_{e}}{q_{max}}$$

where *q_max_* is the maximum adsorption capacity (mg/g), and *K_L_* is the *Langmuir* isotherm constant (L/mg).

The *Freundlich* isotherm is described by Equation (13) [48]:

(13) $$lnq_{e}=lnK_{F}+\frac{1}{n}\cdot lnC_{e}$$

where *K_F_* is the constant of the *Freundlich* isotherm (L/g), and n is the *Freundlich* exponent.

For the *Langmuir* equation, the efficiency of the adsorption process can be predicted by the dimensionless equilibrium parameter *R_L_*, which was defined by the following equation:

(14) $$R_{L}=\frac{1}{1+K_{L}\cdot C_{0}}$$

When 0 < *R_L_* < 1, the adsorption progress is considered as favorable.

## 4. Conclusions

In this work, the SS/CMCS hydrogel with porous structure was prepared by electrodeposition at 3 V DC voltage. SS was confirmed as the major component of the SS/CMCS hydrogel by the Bradford assay (77.12 ± 3.48%). The interaction between SS and CMCS molecules in the SS/CMCS hydrogel was confirmed by FTIR and viscosity measurements as intermolecular hydrogen bonds. Further, the SC samples were prepared by the carbonization method at 850, 950 and 1050 °C. The results of N_2_ adsorption–desorption, SEM, and Raman spectra displayed that the SC1050 had a larger specific area (up to 1494.96 cm^2^/g), more developed pore structure, and higher graphitization degree.

While many factors affected the adsorption process, such as initial MB concentration, solution’s initial pH, adsorption time, and temperature, the dye adsorption experiments demonstrated that the SC1050 had the best adsorption capacity. The adsorption of MB by SC1050 followed the intra-particle diffusion model. The whole adsorption process was exothermic and fitted well to the *Langmuir* isotherm. The optimum adsorption condition is T = 15 °C, pH = 10, and the maximum monolayer adsorption amount is 231.79 mg/g. Those results demonstrated that the SC1050 could be used as biological adsorbents for MB. 

Our work is expected to provide a more energy-saving and efficient approach for the enrichment and recovery of SS from wastewater of the silk industry. In addition, the SS-derived carbonous adsorbent demonstrates good adsorption capability to MB and, therefore, has good potential application prospects in the field of dye wastewater treatment. 

## Figures and Tables

**Table 1:** Viscosity.

Samples	Viscosity (mpa·s)
8% SS solution	1.82 ± 0.02
1% CMCS solution	5.04 ± 0.02
4% SS/0.5% CMCS mixture	3.72 ± 0.01

**Table 2:** The average pore sizes of SS/CMCS hydrogel, SC850, SC950 and SC1050.

Samples	Pore Size (μm)
SS/CMCS	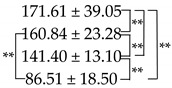
SC850	
SC950	
SC1050	

(n = 30, ** *p* < 0.01).

**Table 3:** Microstructural information of samples SC850, SC950 and SC1050.

Samples	BET Surface Area (cm^2^/g)	BJH Average Pore Diameter (nm)	Total Pore Volume (cm^3^/g)
SC850	566.07	2.94	0.05
SC950	713.66	2.79	0.07
SC1050	1494.96	2.63	0.54

**Table 4:** Thermodynamic parameters for the adsorption of MB onto SC1050.

T (K)	Δ	∆*H* (kJ/mol)	$\Delta S\;(J\cdot \mathrm{mol}{}^{-1}\cdot K{}^{-1})$
288.15	−0.98 ± 0.53	−5.01 ± 0.53	−3.39 ± 1.74
298.15	−1.02 ± 0.74		
308.15	−1.05 ± 0.75		
318.15	−1.08 ± 0.77		

**Table 5:** Kinetic parameters for the adsorption of MB onto SC1050 obtained by plotting.

Sample	Pseudo-First Order			Pseudo-Second Order			Intra-Particle Diffusion		
	q_1_ (mg/g)	k_1_ (1/min)	R^2^ (%)	q_2_ (mg/g)	k_2_ (g·mg^−1^·min^−1^)	R^2^ (%)	k_i_ (mg·g^−1^·min^−1/2^)	C_i_	R^2^ (%)
SC1050	202.21	0.02	98.63	269.54	1.32 × 10^−4^	99.73	21.49	12.85	99.81

**Table 6:** Parameters of adsorption isotherms from *Langmuir* and *Freundlich* models.

Sample	*Langmuir* Isotherm Model			*Freundlich* Isotherm Model		
	R_L_	K_L_ (L/mg)	R^2^ (%)	n	K_F_ (L/mg)	R^2^ (%)
SC1050	0.19	0.52	99.99	17.89	1.64	81.98

## Data Availability

Data are contained within the article and Supplementary Materials.

## Supplementary Materials

The following are available online at https://www.mdpi.com/article/10.3390/ijms23031668/s1.
