# Supplementary material for: Silk Sericin Enrichment through Electrodeposition and Carbonous Materials for the Removal of Methylene Blue from Aqueous Solution
Source: Int J Mol Sci. 2022 Jan 31;23(3):1668. doi: 10.3390/ijms23031668 (PMC8836085; doi:10.3390/ijms23031668)
Supplement: Supplementary file 1 [file ijms-23-01668-s001.zip › ijms-1551442-supplementary.pdf]

Supplementary Materials

Silk sericin enrichment through electrodeposition and carbonous materials for the removal of methylene blue from aqueous solution

Yansong Ji [1], Xiaoning Zhang [1,*], Zhenyu Chen [1], Yuting Xiao [1], Shiwei Li [1], Jie Gu [2], Hongmei Hu [2] and Guotao Cheng [1]

[1] State Key Laboratory of Silkworm Genome Biology, College of Sericulture, Textile and Biomass Sciences, Southwest University, Chongqing 400715, China;
[2] Key Laboratory of Sustainable Utilization of Technology Research for Fisheries Resources of Zhejiang Province, Zhejiang Marine Fisheries Research Institute, Zhoushan 316021, P. R. China;
* Correspondence: xzhang@swu.edu.cn;

Table of Contents

1. Optimization of gelation........S2
1.1. Determination of CMCS concentration........S2
1.2. Determination of driving voltage for gelation........S3
1.3. Determination of electrodeposition time........S4
2. The presumed gelation mechanism........S4
3. Determining the weight percentage of SS in SS/CMCS hydrogel........S5
4. Raman spectra with the deconvolution of D and G peaks........S6
5. Cell morphology........S6
6. Removal of MB from aqueous solution by SC1050........S7
7. Determination of the pH_{pzc} of SC1050........S8
8. UV-Vis spectra of MB at different pH values........S8
9. References........S9

1. Optimization of gelation parameters

1.1. Determination of CMCS concentration

The sericin content in silk cocoon is 20 - 30% [1] and the silk sericin (SS) concentration in the wastewater varies depends on the degumming technique applied and the usage of silk after the degumming process [2-6]. We believe 4% (w/v) is an easy-to-achieve SS concentration from the degumming wastewater, either via dilution or condensation, and 4% (w/v) of SS solution was selected for the gel preparation.

We then investigated the effect of CMCS concentration on the gelation. As shown in Figure S1a, when the CMCS concentration was 0.1% (w/v), a gel-like sludge can be found floating on the surface of the solution. The newly generated hydrogel cannot be captured by the electrode due to the weak attraction force resulting from the low concentration of negatively charged CMCS molecules within the gel. In addition, the hydrophilicity of silk sericin would drag the hydrogel into solution.

When the CMCS concentration increased to 0.2% (w/v), the contour of the hydrogel in the solution can be clearly seen (Figure S1b). As the CMCS concentration continue to rise, the thickness of the hydrogel increased. In addition, although the generated hydrogel remains transparent, its color gradually became darker with the increase of CMCS concentration (Figure S1c-e), indicating the formation of the denser gel structure.

Furthermore, it was found that the hydrogel generated with CMCS concentrations of 0.1%-0.4% (w/v) would slide off from the graphite electrode when the electrode was removed from the SS/CMCS mixture, as Figure S1f-k depicted, which was quite unfavorable for the separation of hydrogel from liquid phase. In contrast, the hydrogel generated at the CMCS concentration of 0.5% (w/v) was well attached to the graphite electrode and can be separated from the SS/CMCS mixture easily (Figure S1l). Therefore, the CMCS concentration of 0.5% (w/v) was selected for the preparation of SS/CMCS hydrogel.

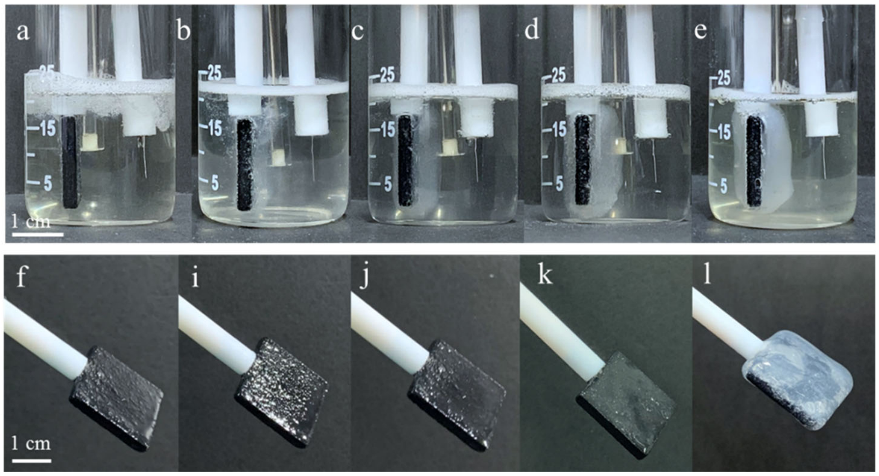

Figure S1. The effect of CMCS concentration on the gelation, the concentration of CMCS is (a) 0.1% (w/v), (b) 0.2% (w/v), (c) 0.3% (w/v), (d) 0.4% (w/v), (e) 0.5% (w/v); The electrodes in (f)-(l) corresponds to those in (a)-(e) respectively, and the photos were captured once the electrodes were lifted from the SS/CMCS mixture, after the gelation.

1.2. Determination of driving voltage for gelation

The effect of driving voltage on the gelation of 4% SS/ 0.5%CMCS solution was explored. As shown in Figure S2a, no hydrogel can be observed on the surface of graphite electrode at 1 V of driving voltage, while a thin layer of hydrogel can be found on the top of the graphite electrode at 2 V of driving voltage (Figure S2b). When the driving voltage was increased to 3 V (Figure S2c), the formation of a thick and dense hydrogel can be observed on the surface of the graphite electrode. Therefore, 3 V was selected as the driving voltage for the preparation of SS/CMCS hydrogel.

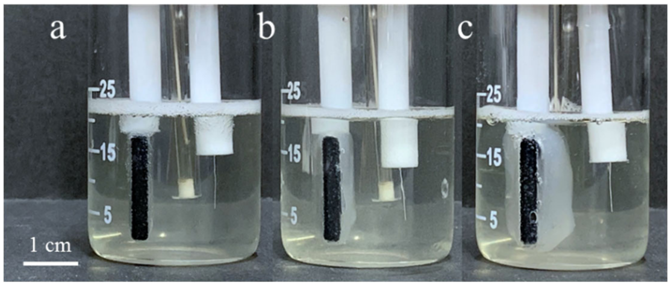

Figure S2. The effect of driving voltage on the gelation: (a) 1 V, (b) 2 V, (c) 3 V.

1.3. Determination of electrodeposition time

The effects of electrodeposition time (1 min, 3 min, 5 min, and 7 min) on the gelation was explored. As shown in Figure S3, the SS/CMCS hydrogel became thicker as the electrodeposition time increased. However, once the electrodeposition time was increased to 7 min (Fig. S3d), the hydrogel generated on the surface of graphite electrode had already touched the platinum electrode, which may have affected the structure and properties of the hydrogel fabricated. Therefore, 5 min of electrodeposition time was selected for the preparation of SS/CMCS hydrogel.

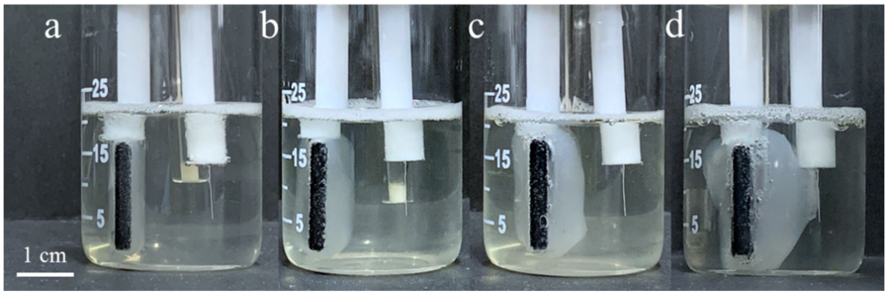

Figure S3. The effect of electrodeposition time on the gelation: (a) 1 min, (b) 3 min, (c) 5 min, and (d) 7 min.

2. The presumed gelation mechanism

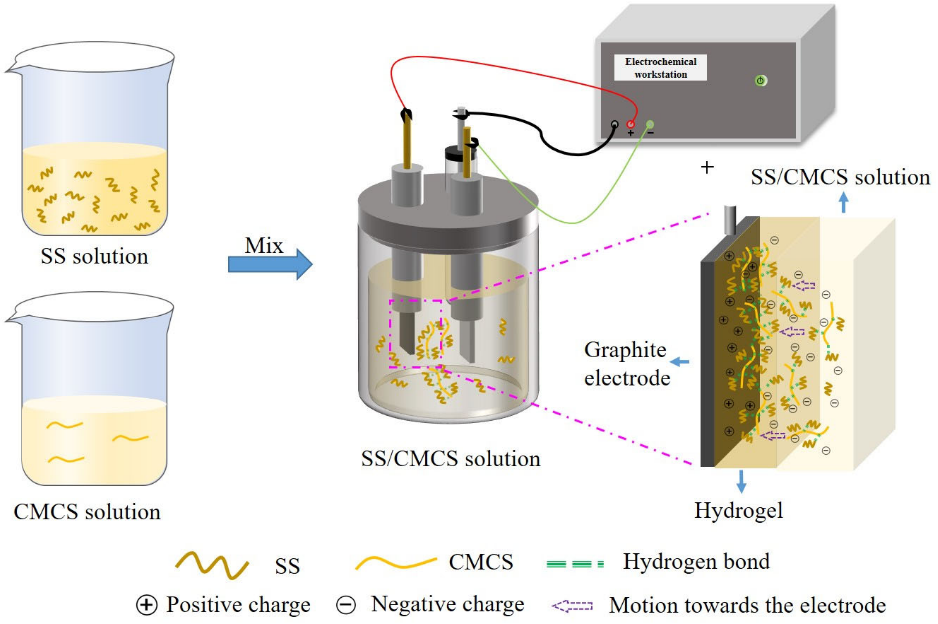

Figure S4. The proposed mechanism of the gelation process.

3. Determining the weight percentage of SS in SS/CMCS hydrogel

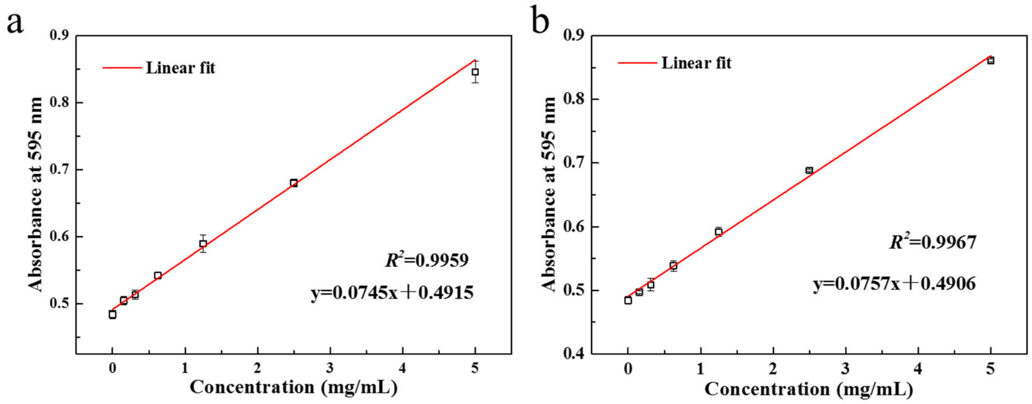

Figure S5. The standard curves of SS content in SS/CMCS mixture (a) before and (b) after gelation.

Table S1. Measurement of SS content in SS/CMCS hydrogel

Sample number	Before gelation		After gelation		Weight of hydrogel (mg)	SS content (%)
	SS concentration (mg/mL)	Volume (mL)	SS concentration (mg/mL)	Volume (mL)		
**1**	38.87	20	36.84	16.20	222.98	80.99
**2**	38.70	20	37.45	16.10	230.38	74.25
**3**	38.49	20	37.20	15.80	239.12	76.13
**Average**	38.69	20	37.16	16.03	230.83	77.12
**Standrad deviation**	0.19	0	0.31	0.21	8.08	3.48

4. Raman spectra with the deconvolution of D and G peaks

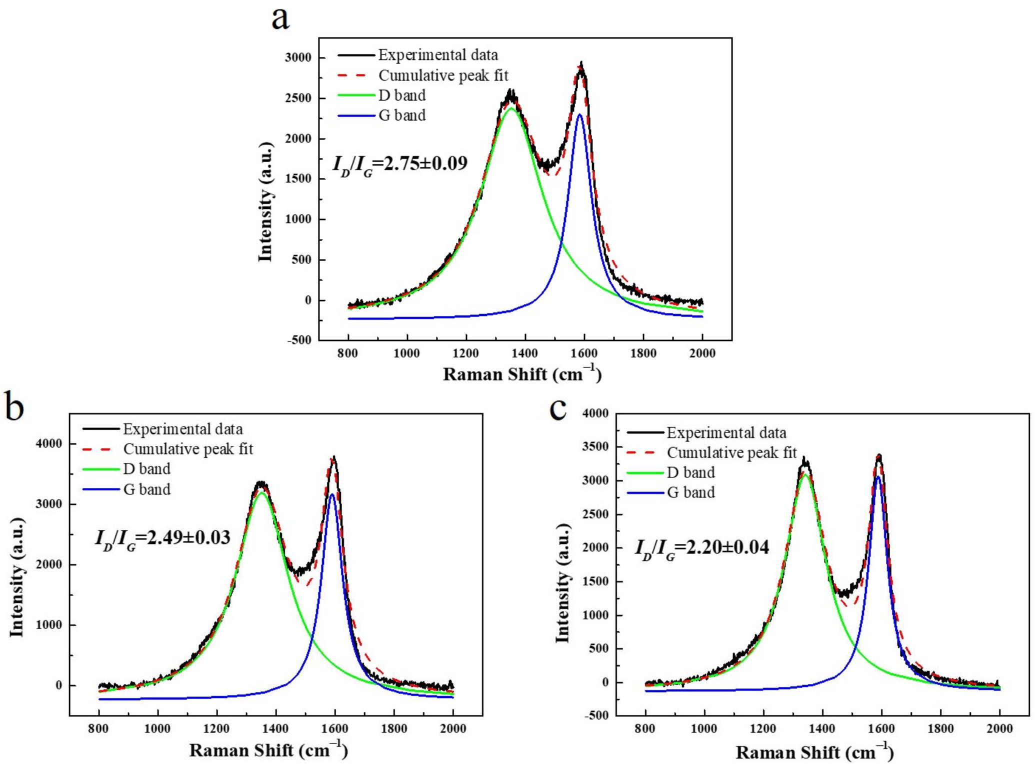

Figure S6. Deconvoluted Raman spectra of samples (a) SC850, (b) SC950 and (c) SC1050.

5. Cell morphology

As shown in Figure S7, the cells in the SC1050 group and the control group were spindle or polygonal shape, and the cell density gradually increased over time, indicating the cells were successfully proliferated. On the contrary, cells cultured in 64 g/L of phenol solution adopt spherical shape with limited number of cells.

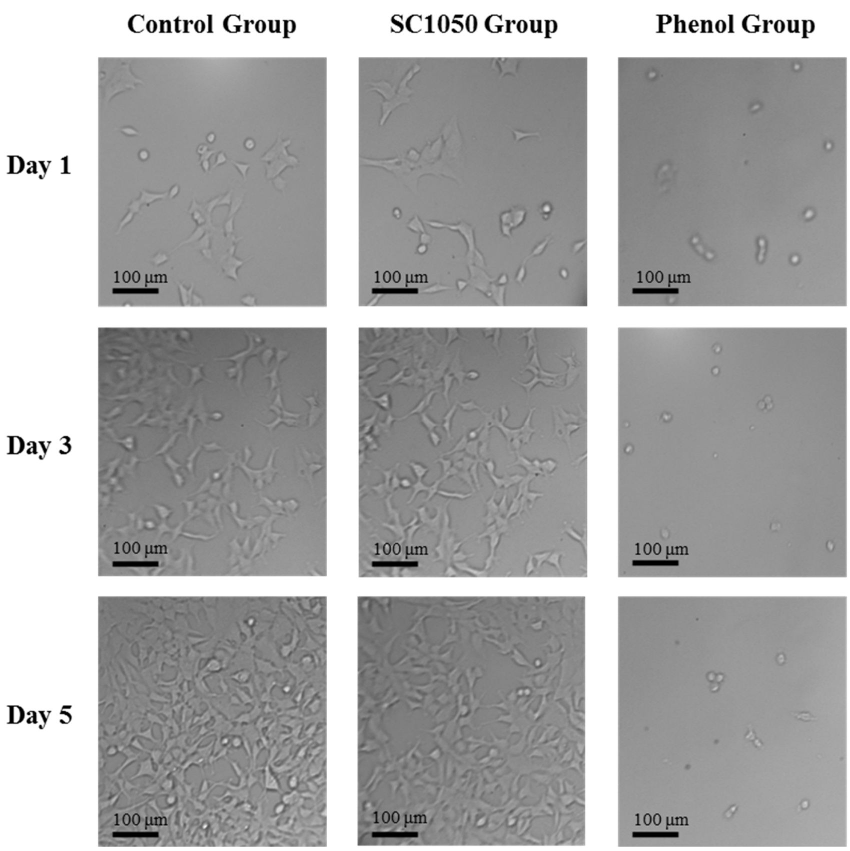

Figure S7. HEK293 cell morphology for control, SC1050 and phenol groups of the CCK-8 assay on day 1, day 3, and day 5.

6. Removal of MB from aqueous solution by SC1050

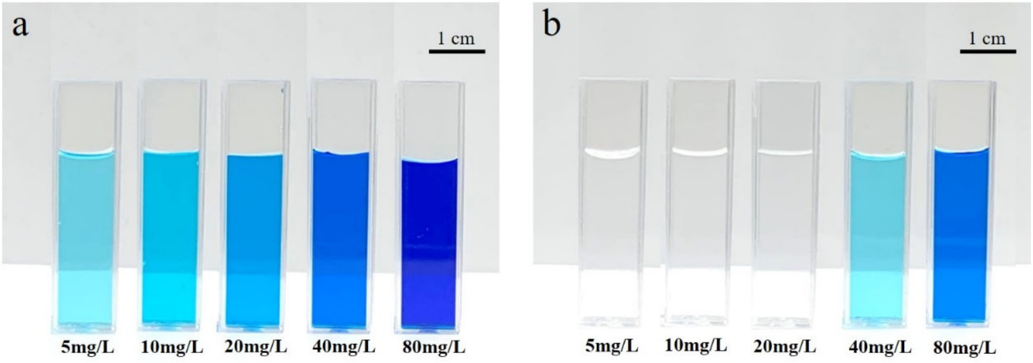

Figure S8. Photographs of MB solutions (a) before and (b) after adsorption by SC1050.

7. Determination of the pH_{pzc} of SC1050

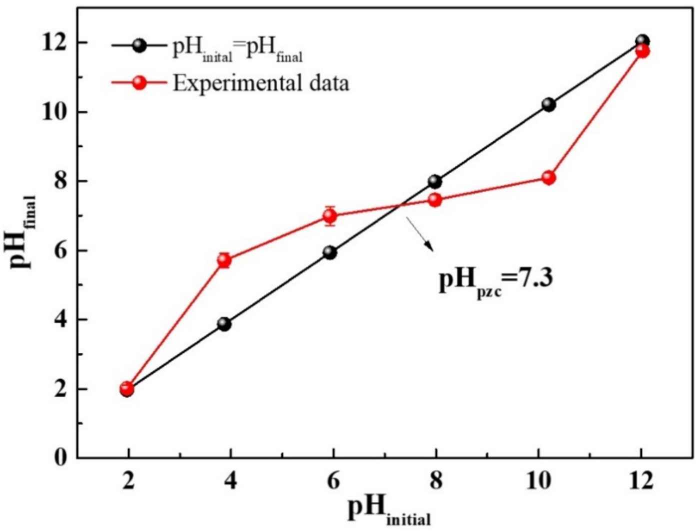

Figure S9. pH_{pzc} of SC1050.

8. UV-Vis spectra of MB at different pH values

As shown in Figure S10, methylene blue, in the solution with pH values range from 2 to 10, has two absorbance peaks at 664 nm and 617 nm, respectively, and the maximum absorbance peak was observed at 664 nm. Such observation was consistent with the previous reports [7; 8]. However, the maximum absorbance peak shifted from 664 nm to 617 nm under the strongly alkaline condition (pH = 12), which can be attributed to the conversion of methylene blue into methylene violet [7; 9].

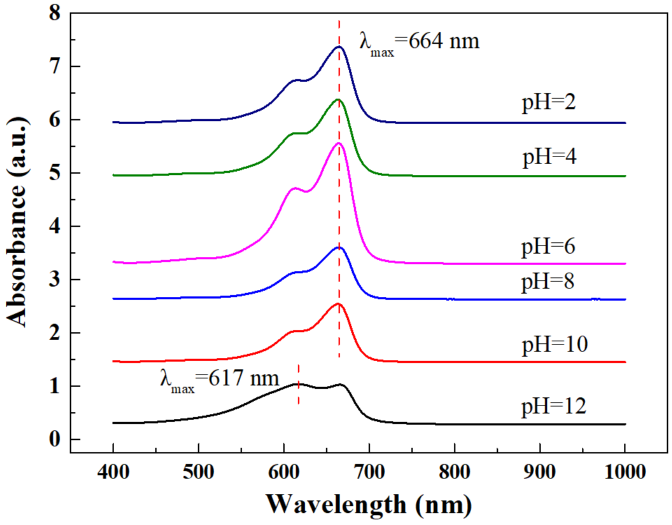

Figure S10. UV-Vis spectra of MB at different pH values.

9. References

1. Aramwit, P.; Napavichayanun, S.; Pienpinijtham, P.; Rasmi, Y. Antibiofilm activity and cytotoxicity of silk sericin against *Streptococcus* mutans bacteria in biofilm: an *in vitro* study. *J. Wound Care* **2020,** *29,* S25-S35. https://doi.org/10.12968/jowc.2020.29.Sup4.S25.
2. Capar, G.; Aygun, S.S.; Gecit, M.R. Separation of sericin from fatty acids towards its recovery from silk degumming wastewaters. *J. Membr. Sci.* **2009,** *342,* 179-89. https://doi.org/10.1016/j.memsci.2009.06.039.
3. Vaithanomsat, P.; Kitpreechavanich, V. Sericin separation from silk degumming wastewater. *Sep. Purif. Technol.* **2008,** *59,* 129-33. https://doi.org/10.1016/j.seppur.2007.05.039.
4. Lin, J.X.; Wang, L. Coagulation of sericin protein in silk degumming wastewater using quaternized chitosan. *J. Polym. Environ.* **2012,** *20,* 858-64. https://doi.org/10.1111/j.1751-1097.1987.tb05360.x.
5. Zhao, Z.L.; Li, W.W.; Wang, F.; Zhang, Y.Q. Using of hydrated lime water as a novel degumming agent of silk and sericin recycling from wastewater. *J. Clean. Prod.* **2018,** *172,* 2090-6. https://doi.org/10.1016/j.jclepro.2017.11.213.
6. Gupta, D.; Agrawal, A.; Rangi, A. Extraction and characterization of silk sericin. *Indian J. Fibre. Text. Res.* **2014,** *39,* 364-72.
7. Adamčíková, L.; Pavlíková, K.; Ševčík, P. The decay of methylene blue in alkaline solution. *React. Kinet. Catal. Lett.* **2000,** *69,* 91-4. https://doi.org/10.1023/A:1005696926749.
8. Ohline, S.M.; Lee, S.Y.; Williams, S.; Chang, C.N. Quantification of methylene blue aggregation on a fused silica surface and resolution of individual absorbance spectra. *Chem. Phys. Lett.* **2001,** *346,* 9-15. https://doi.org/10.1016/S0009-2614(01)00962-9.
9. Kelly, J.M.; Van Der Putten, W.J.M.; Mcconnell, D.J. Laser flash spectroscopy of methylene blue with nucleic acids. Effects of ionic strength and ph. *Photochem. Photobiol* **2010,** *45,* 167-75. https://doi.org/10.1111/j.1751-1097.1987.tb05360.x.
